# Moxibustion for treating knee osteoarthritis: A protocol for systematic review and meta analysis

**DOI:** 10.1097/MD.0000000000019974

**Published:** 2020-05-08

**Authors:** Xue Wang, Yunfeng Jiang, Jun Xiong, Ting Yuan, Jun Yang, Xiaohong Zhou, Kai Liao, Lingling Xu

**Affiliations:** a Jiangxi University of Traditional Chinese Medicine; b Affiliated Hospital of Jiangxi University of Traditional Chinese Medicine, Nanchang, PR China.

**Keywords:** knee osteoarthritis, moxibustion, overview, systematic reviews

## Abstract

**Background:**

Knee osteoarthritis (KOA) is a significant health issue because it causes pain and functional limitation. Many studies have reported that moxibustion, a treatment in traditional Chinese medicine, is effective in treating KOA. The aim of this overview is to synthesize and assess the reliability of evidence generated from these systematic reviews of the effectiveness of moxibusition for KOA.

**Methods:**

This is a protocol for a systematic overview of reviews. We will search 7 databases: PubMed, Embase, Cochrane Library, Chinese Biomedical Literatures Database, China National Knowledge Infrastructure, WangFang Database, Chinese Scientific Journal Database from their inception to April 2020. We will consider systematic reviews and meta-analysis of randomized controlled trials evaluating the effectiveness of moxibustion for KOA. Independent reviewers will sift, perform data extraction in duplicate, and assess the quality of the reviews using the Change MeaSurement Tool to Assess Systematic Reviews-2 to Assessment of Multiple Systematic Reviews-2 and the Preferred Reporting Item for Systematic Review and Meta-Analysis statement. The outcomes of interest include: quality of life, knee function, and pain relief outcomes prioritized in the individual reviews. The evidence will be synthesized where appropriate by patient subgroups, intervention type, context, and outcome. Revman 5.3 software will be used to conduct meta-analysis and calculate the risk ratio for dichotomous data. Weighted mean difference or standard mean difference will be calculated for continuous data. The quality of evidence was assessed according to Grades of Recommendation, Assessment, Development, and Evaluation (GRADE) considering the methodological quality of the randomized controlled trials and meta-analysis.

**Results:**

The results of this study will be published in a peer-reviewed journal.

**Conclusions:**

We expect to compile evidence from multiple systematic reviews of symptomatic improvement in patients with KOA in an accessible and useful document.

**Trial registration number:**

CRD42019141029 in PROSPERO 2019.

## 1 Introduction

Knee osteoarthritis (KOA), known as severe degenerative arthritis, is common in middle-aged and elderly people. It causes pain and dysfunction that greatly reduce the patients’ quality of life. ^[1]^ The epidemiology of the osteoarthritis is complex and multifactorial, with genetic, biological, and biomechanical components.^[2]^ It was reported that the total prevalence is about 15%, 10% to 17% for people over 40, 50% for people over 60, and 80% for people over 75 in China.^[3]^ Many epidemiological studies have shown that women with KOA are more than men, especially postmenopausal women. The rapid increase in the incidence of postmenopausal KOA in women suggests that the lack of sex hormones, especially estrogen, may be a systemic predisposing factor for KOA.^[4,5]^ With the coming of the aging of the world population, KOA has become a major public health problem threatening the health of the elderly and affecting the quality of life, and has become the second major disability disease after cardiovascular disease.^[6]^ Western medicine treatment of KOA mostly adopts basic treatment, such as health education, non steroidal anti-inflammatory drugs, pain relief, intra-articular injection of medical sodium hyaluronate, and other measures to improve the pain and clinical symptoms of patients.^[7]^ The final outcome is to take surgical methods to solve the problem. The above treatment plans have their own disadvantages. Drug treatment caused adverse reactions such as kidney and gastrointestinal tract. The loosening of prosthesis, service life, and rejection of the body in the later stage of operation plan brought troubles to patients.^[8]^ Therefore, many patients with KOA are seeking complementary and alternative treatments.^[9]^

Traditional Chinese medicine (TCM) believes that KOA belongs to the category of “arthralgia of bone” and “arthralgia of knee”, which are mostly caused by old age and frailty, liver and kidney deficiency, insufficient blood and qi, loss of tendons and veins, and exogenous wind, cold, and dampness.^[10]^ Moxibustion is a TCM therapy suitable for some chronic and severe diseases and works by stimulating acupuncture points with thermal energy from ignited moxa. Moxibustion takes advantage of the characteristics of green therapy. Moxa leaves have a scented aroma, slightly hot nature, and pure yang. It has the effects of activating collaterals, regulating qi, and blood.^[11]^ At present, it has been found that the physiological and pathological mechanism of osteoarthritis is complex. It is believed that it is the result of the joint action of a variety of inflammatory factors, among which one of the key factors is interleukin and tumor necrosis factor.^[12]^ A recent study showed that heat-sensitive moxibustion can inhibit the expression of interleukin-1 beta, tumor necrosis factor-α, and matrix metalloproteinases 13, reduce the inflammatory reaction, and alleviate the joint damage.^[13]^ Studies have shown that moxibustion may reduce inflammatory reaction and protect articular cartilage by inhibiting the expression of NO (nitric oxide) and a disintegrin and metalloproteinase with thrombospondin motif-4 .^[14]^ Published studies on moxibustion for the treatment of KOA describe diverse clinical applications, with most types of moxibustion for KOA demonstrating positive effects.^[15–19]^

Recently, several systematic reviews (SRs)^[20–24]^ have reported the effectiveness of moxibustion on pain relief and functional recovery in patients with KOA. Nevertheless, the evidence for the effectiveness of moxibustion as the treatment for KOA has not been thoroughly evaluated yet. Therefore, we conducted this overview of moxibusition as intervention for KOA patients, critically appraised and synthesized the results from these SRs in order to provide more reliable evidence-based medical references for clinical practitioners and researchers.

## 2 Methods and analysis

### 2.1 Protocol and registration

This protocol was designed in accordance with the methodological guidelines for overviews provided by the Cochrane Collaboration, the Joanna Briggs Institute, and the Preferred Reporting Items for Systematic Reviews and Meta-analysis Protocols (PRISMA-P; checklist provided). It is registered on the International Prospective Register of Systematic Reviews (PROSPERO no. CRD42019141029; https://www.crd.york.ac.uk/prospero/display_record.php?RecordID=141029). Ethics approval is not required for this review as we will analyze published literature only.

### 2.2 Eligibility criteria

The SRs of moxibustion for KOA met the inclusion criteria as following were included.

#### 2.2.1 Types of studies

SRs of randomized controlled trials (RCTs) were included, in which moxibistion was utilized as the treatment for KOA.

#### 2.2.2 Types of participants

Participants who have been diagnosed as KOA. There were no restrictions on gender, age, or race.

#### 2.2.3 Types of interventions

SRs that involved any form of moxibustion (e.g., direct moxibustion, indirect moxibustion, heat-sensitive moxibustion, moxa burner moxibustion, warm needling, crude drug moxibustion, or natural moxibustion) as the sole treatment or as a part of a combination therapy with another intervention (e.g., conventional drugs) will be included.

#### 2.2.4 Type of comparator (s)/control

There is no limit to the treatment of the control group, including no treatment, or placebo, or any control considered for comparison in the individual system review.

#### 2.2.5 Types of outcome measures

##### 2.2.5.1 Primary outcomes

Osteoarthritis index evaluation using Western Ontario and McMaster Universities Osteoarthritis Index (WOMAC).

##### 2.2.5.2 Secondary outcomes

Secondary outcome measures will include:

1. Quality of life: using the MOS item short from health survey (SF-36).
2. Pain relief: Visual Analogue Scale, Numerical Rating Scale.
3. Function measures: Lysholm knee scoring scale.

### 2.3 Search methods for identification of studies

We will search 7 databases: PubMed, Cochrane Database of Systematic Reviews (Cochrane Library, Wiley), Embase, Chinese Biomedical Literatures Database, China National Knowledge Infrastructure, WangFang Database, Chinese Scientific Journal Database from their inception to April 2020. The initial search strategy (Table 1) was developed for the PubMed database using subject headings and free-text words that describe moxibustion for KOA. Search strategies for the other databases will be adapted as necessary. No date or language restrictions will be placed on our search, however, there may be restrictions due to the criteria within the individual reviews. The following search terms will be used: knee osteoarthritis, degenerative arthritis, osteoarthritis, senile osteoarthritis, genual osteoarthritis, hypertrophic arthritis, moxibustion, thunder fire miraculous moxa roll, thunder fire moxibustion, taiyi miraculous moxa roll, suspended moxibustion, mild moxibustion, needle warming moxibustion, SR, meta-analysis, network meta-analysis etc.

**Table 1:**
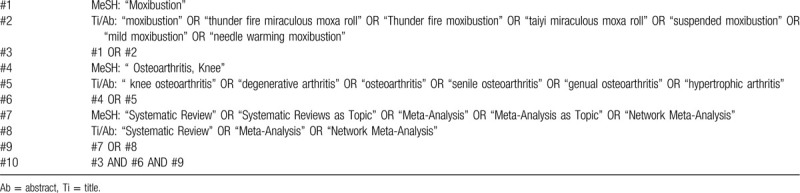
Search Strategy (PubMed).

### 2.4 Selection of studies

**Figure 1:**
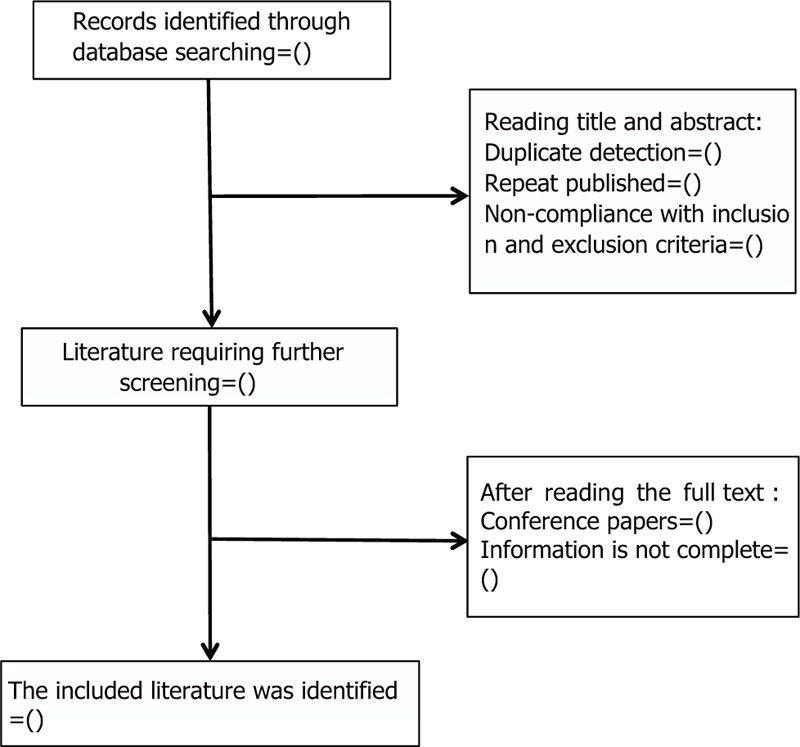
Flowchart of literature selection.

Two authors (WX, YT) will independently screen titles and abstracts to identify relevant studies for full-text review and will independently screen full texts for final inclusion, which is based on the eligibility criteria. Two reviewers will independently perform review selection with the full-text screening questionnaire, which includes the following reasons for exclusion: not a SR, does not summarize RCT data separately, conference abstract with insufficient data. We will not contact authors for clarification. We will resolve any disagreements regarding the inclusion or exclusion of individual reviews by discussion with a third reviewer (YJ). The entire process of selecting studies is shown in the PRISMA flow diagram (Fig. 1). This will result in a final list of included and excluded systematic reviews along with reasons for exclusion. This process will not be blinded so all reviewers will be able to see the authors and their affiliated institutions.

### 2.5 Data extraction and management

Search results will be exported into NoteExpress 3.2.0 for duplication. The full text of reviews shortlisted for full-text analysis will also be uploaded to NoteExpress 3.2.0. We shall perform data extraction using Microsoft Excel.

A standardized data extraction form was designed in advance. After identifying all the eligible SRs, 2 authors (XHZ and LLX) independently extracted data according to data extraction form and then cross-checked. Information such as year of publication, number of patients enrolled, participant characteristics, features of interventions in treatment and control groups, types of outcome assessment, methodological quality of primary studies, data analysis approaches, and sources of funding were extracted. Discrepancies in the data extracted will be resolved by discussion and reaching a consensus, and if necessary, arbitration by a third author (KL). When the data was incomplete, the reviewers tracked back to the primary studies of included SRs.

### 2.6 Assessment of methodological quality of included reviews

Two authors (XW and YFJ) will independently assess the quality of each review using the AMSTAR-2 tool.^[25]^ This is most commonly used to assess the quality of SRs included in overviews. AMSTAR-2 is an update of AMSTAR, which can be used to appraise SRs of both randomized and non-randomized controlled trials. AMSTAR-2 includes 16 items, with each of the 16 criteria given a rating of “yes” (definitely done), “no” (definitely not done), “can’t report” (unclear if completed), or “not applicable” based on information provided by the SRs on which reviewers put an evaluation when the criterion is met. Discrepancies in the ratings of the methodological reviews will be resolved by consensus between the authors and, if necessary, arbitration by a third author (JY).

### 2.7 Assessment of reporting quality of included reviews

Two authors (TY and KL) evaluated the reporting quality of the included SRs independently by using the PRISMA. Consensus was reached by discussion between 2 reviewers or an independent decision obtained from the third author (XW), if necessary.

The PRISMA statement^[26]^ for reporting quality consists of a 27-item checklist and a 4-phase flow diagram. The checklist includes items deemed essential for transparent reporting of a SR. Each item of the PRISMA form was graded as “yes,” “incomplete,” or “no”. The sum of all items scored for each questionnaire was divided by its maximum possible score to assess study quality as a percentage.

### 2.8 Assessment of the quality of the evidence in reviews

We will aim to extract GRADE^[27]^ ratings from each included review. Similar to previous overviews, we will make judgments to downgrade or upgrade the quality of evidence based on the risk of bias using criteria specified by the GRADE working group. This re-evaluation will only be conducted on reviews included in the finally hierarchical index of interventions. Discrepancies in the ratings of the quality of evidence will be resolved by consensus between the authors and, if necessary, arbitration by a third author (XW).

### 2.9 Data synthesis

We will consider the issue of overlapping primary studies prior to preparing our evidence synthesis. If there are multiple SRs of the same intervention in the same patient population, and for the same outcome, we will apply the following:

1. If the primary studies are completely overlapping, then we will select the highest-quality review.
2. If the primary studies partially overlap, then we will retain both reviews if the lower-quality review consists of more than one-third new studies.
3. If the primary studies do not overlap, then we will retain both reviews.

We will extract the data from the original study from the SR and use Cochrane Collaboration's Windows software (Review Manager (Rev Man) V.5.3) for all statistical analysis. Statistical heterogeneity between studies was assessed by the χ^2^ test with a *P* value of ≤.10 indicating significant heterogeneity. The magnitude of heterogeneity was categorized by the I^2^ statistic with I^2^ of 0% to 24% = no heterogeneity, I^2^ of 25% to 49% = moderate heterogeneity, I^2^ of 50% to 74% = substantial heterogeneity, and I^2^ of 75% to 100% = considerable heterogeneity.

### 2.10 Subgroup analysis

If the necessary data are available, subgroup analysis will be carried out according to different factors as follows:

1. Control interventions (e.g., sham/placebo moxibustion, no treatment, other TCM treatment or non-TCM treatment).
2. Type of moxibustion (e.g., heat-sensitive moxibustion, thunder fire miraculous moxa roll, warm needling moxibustion, suspended moxibustion or mild moxibustion).

## 3 Discussion

This overview will be the first summary of existing systematic reviews aimed to summary the evidence of moxibustion treatment of KOA. The conclusions will be based on types of interventions and finding of the included reviews. In the discussion section in the full report of our study, we plan to include the following subsections, typical for this type of study:

(1) summary of main findings;
(2) strength and limitations;
(3) comparison with other studies and opinions;
(4) interpretation of results; and
(5) conclusion.

This overview, therefore, will help medical workers to implement the most effective interventions to treatment with KOA, and provide patients, doctors, and clinical researchers with information about the credibility of current evidence and research direction in the future.

## Author contributions

**Conceptualization:** Xue Wang, Jun Xiong, Jun yang.

**Data curation:** Jun Yang, XiaoHong Zhou, Ting Yuan.

**Formal analysis:** Kai Liao, LingLing Xu.

**Investigation:** Jun Xiong, Xue Wang, YunFeng Jiang.

**Methodology:** Xue Wang, Ting Yuan, Jun Yang.

**Software:** XiaoHong Zhou, LingLing Xu.

**Supervision:** Jun Xiong, YunFeng Jiang.

**Writing – original draft:** Jun Xiong, Xue Wang, Ting Yuan, Jun Yang.

**Writing – review and editing:** YunFeng Jiang, XiaoHong Zhou, LingLing Xu, Kai Liao.
